# An analysis of self-ignition of mine waste dumps in terms of environmental protection in industrial areas in Poland

**DOI:** 10.1038/s41598-021-88470-7

**Published:** 2021-04-23

**Authors:** Adam Smoliński, Václav Dombek, Eva Pertile, Leszek Drobek, Krzysztof Gogola, Sylwia W. Żechowska, Małgorzata Magdziarczyk

**Affiliations:** 1 Central Mining Institute, Pl. Gwarków 1, 40-166 Katowice, Poland; 2 CEET, Nanotechnology Center, VŠB – TU Ostrava, 17. listopadu 15, 708 33 Ostrava, Poruba, Czech Republic; 3 Department of Environmental Monitoring, Central Mining Institute, Pl. Gwarków 1, 40-166 Katowice, Poland; 4 Department of Economics, Finance, Regional and International Research, Opole University of Technology, ul. Luboszycka 7, 45-036 Opole, Poland

**Keywords:** Ecology, Environmental sciences, Natural hazards

## Abstract

The aim of the paper was to work out a new comprehensive methodology to monitor thermal activity at mine waste dumps. The methodology was tested through monitoring thermal phenomena occurring in the areas of extractive waste dumping facilities located in the Upper Silesian Coal Basin, Poland. Within the framework of the study, a comparative analysis of three waste dumps was performed; the first two of them, which were not previously reclaimed, are in part thermally active, whereas the third one comprises one section which was partially reclaimed and another section which is still being operated. The research objective was to observe the changes of atmospheric emissions of Polycyclic Aromatic Hydrocarbons (PAHs) from the three selected facilities within the period of 21 months of constant monitoring. The novelty of the methodology of thermal activity monitoring at burning mine waste dumps consisted in the application advanced chemometrics methods. The collected data were analyzed by means of the Hierarchical Clustering Analysis supplemented with a color map of the experimental results. Based on the newly developed methodology, it was determined that thermal processes occur in all of the three analyzed sites. The non-reclaimed waste dumps characterize of intense thermal phenomena covering the majority of the studied area. It was also observed that the most intensive thermal activity occurs in the central sections of the dumps with temperature values reaching the level of 600 °C accompanied by high emissions of PAHs. In addition, the research results demonstrate that despite the reclamation processes, there are certain areas which still remain thermally active in one of the studied extractive waste dumps. This manifested itself by high measured concentrations of all the analyzed PAHs and locally increased surface temperatures which, however, did not exceed 200 °C; the majority of the areas of the reclaimed waste dump characterized of temperatures in the range of 20–30 °C.

## Introduction

Waste which is generated in the process of hard coal extraction constitutes a mixture composed of the minerals co-occurring in the seams of bituminous coal and bituminous coal which was not fully recovered in the enrichment of the extracted raw material taking place in mechanical processing plants^1–3^. Mining waste originates from all stages of mine development and extraction activities including shaft sinking, seams opening and finishing as well as all technological operations connected with the enriching and purification processes of the extracted raw material^4,5^. Two major types of extractive waste can be distinguished, namely the mining waste and the coal processing waste^2,6^. Mining waste produced during the opening up phase is characterized by a large variability of petrographic content. The granulation of the rock material is within the range of 0–500 mm. The content of combustible substance in this kind of waste differs depending on the scope and character of the mining activities.

Mining activity has a strong negative impact on the natural environment. Coal mine waste dumps are mostly composed of claystones, mudstones, sandstones, conglomerates, carbonates, carbonaceous shales, and pyrite-bearing carbonaceous rocks^7,8^. The thermal activity of mine waste dumps is characterized by high temperatures inside the dump and also on the surface. Often, an exhalation zone is observed on the surface of the mine waste dump^9,10^. It is a source of the emission of exhaust gases composed of inorganic compounds such as carbon dioxide and monoxide, sulfur dioxide, hydrogen sulfide in addition to organic compounds, e.g. polycyclic aromatic hydrocarbons, phenols, Benzene, Toluene, Ethylbenzene, and Xylene^11,12^.

According to the Central Statistical Office, the annual volume of extractive waste generated in Poland totals 30 million Mg, out of which more than 90% comes from Silesia, Lublin and Malopolskie Provinces where coal the mining sector is concentrated. Despite the fact that approximately 90% of extractive waste generated in Poland is recycled, in most cases it is disposed of on ground surface in earthworks, earth structures, land levelling, engineering works and the filling of adversely transformed lands^7^. The distinction between the disposal and the recycling is made only from the legal point of view because, technically, there are no major differences between the recycling and disposal of extractive waste. The differences occur only in terms of the morphology and the required geotechnical parameters of the waste dumps as well as the designation of the dumping site^6^. Taking into consideration the above differences, the following basic forms of extractive waste disposal on the ground surface can be distinguished^3,4^:

- disposal in extractive waste dumping facilities, in the past in bings and spoil piles;
- filling excavation voids in open cast mining;
- reclamation of landfills and degraded areas;
- levelling of subsidence areas and lands affected by underground mining activities;
- regulation of surface watercourse channels and the construction of flood embankments;
- surface hardening, engineering works, transportation embankments, earth works, etc.;
- landscaping (skiing slopes, recreational areas, etc.).

Currently, the volume of post extractive waste in Poland accounts for over 550 million Mg. In the Upper Silesian Coal Basin, there are 152 extractive waste dumping facilities covering a total area of 3800 ha^14,15^.

The most serious potential threat associated with the disposal of extractive waste is the possibility of self-ignition and the resulting endogenous fires of the waste dump^16–19^. The first attempts at establishing the legal regulations concerning the depositing of extractive waste prone to self-ignition were created in the UK in the 1930s. Nowadays, the legal regulations exist in all countries belonging to the European Union.

Until the 1960s, the technologies used for the construction of extractive waste dumps were conducive to the development of thermal phenomena; particularly, in the case of the cone shaped dumps characteristic to that era. Extractive waste was transported to the dump site by means of a conveyor belt and transferred from the top of the waste bank; as a result, the site assumed the shape of a cone^14^. The disposal of the extractive waste did not involve any kind of compacting, and, due to natural slide, the fraction separation process started. Coarse fractions accumulated in the lower sections of the cone, whereas fine fractions remained in the higher ones. With greater heights (up to 100 m) and a considerable inclination of the scarp, the phenomenon of fraction separation contributed to the occurrence of the chimney draft effect, i.e. the aeration of the interior through the loosened lower section of the scarp^21,22^. Consequently, the coal oxidation processes intensified leading to heat accumulation and then to the self-ignition of the extractive waste^23,24^. In the past, fire phenomena took place in the majority of the cone shaped dumps located in Poland^25^.

Another technology which contributed to the development of fire phenomena was the so-called dumping front technology used especially for backfilling the voids in open cast mining and in extractive waste sites covering large areas^26^. Within this technology, extractive waste delivered to the dumping site was relocated by means of bulldozers to the front of the dump and poured from the previously constructed part of the dump towards the front advance. There was a variation of the technology in which the process was performed with the use of stackers. Like in the case of cone shaped dumps, the separation of fractions took place during the dumping, which in turn led to the aeration of the interior. Thick-layered disposal of extractive waste impedes effective compaction of the layers, and, in consequence also stimulates thermal processes. The mechanisms of endogenous fires occurring in extractive waste dumping facilities have been widely discussed in the field literature^19,27,28^. The mechanism consists in a coal oxidation reaction which is an exothermic process taking place even in low temperatures. Coal has a relatively low specific heat capacity and a low thermal conductivity coefficient; therefore, the heat which is emitted during the oxidation process produces a high increase in temperature.

The aim of the paper is to use a new methodology to monitor thermal activity at mine waste dumps. The crucial part of the study was to test this methodology through monitoring the changes in the volumes of gases emitted from extractive waste dumps with thermal activities and then to perform a comparative analysis of the data. The tested facilities were located in the Upper Silesian Coal Basin, Poland. Fire hazards connected with the deposition of extractive waste on the ground surface constitutes a serious problem in Poland. Environmental awareness as well as restrictive EU law enforced the development of new technologies which enable to eliminate fire hazard at mine waste dumps. The first attempts at creating legal regulations concerning the deposition of extractive waste prone to self-ignition were made in Great Britain in the early 1930s. Later, in the 1970s also Germany and France created legal instruments regulating the environmental requirements regarding the handling and management of extractive waste^29,30^. Poland as an EU member state developed appropriate procedures and legal instruments which ensure the standards of ground quality in terms of the excessive contents of chemical elements and substances^31,32^.

## Materials and methods

### Extractive waste dumping facilities

For the purpose of the research, three extractive waste dumps located in the Upper Silesian Coal Basin were selected. The first facility (I) is in part burnt-out but there are also sections which are still thermally active. Dumping facility A contains waste coming from a processing plant, it has been disposed of at the site since the beginning of the twentieth century. The dump is composed of three no longer operated sections, (i) a burnt-out cone with an apex at 406 m above sea level, (ii) the area of a former truncated cone and a flat dump, and (iii) the section where extractive waste from current production is disposed of. The plane of the truncated cone and the flat dump are at 352 m.a.s.l.; the area of the dump totals approximately 34 ha, whereas the estimated cubature of the site is about 15 million m^3^. Thermal processes characterized by various intensities are observed at the facility in addition to clearly visible numerous cracks and fissures emitting gases and water vapor. A subsidence, the so-called crater, which probably appeared in the first half of 2014, is a source of odors and fire gases emissions and constitutes the most problematic area.

The second facility (II) is a no-longer operated, non-reclaimed and in part thermally active extractive waste dump of a 37 ha area. The estimated cubature of the site equals about 3 million m^3^. The extractive waste which was disposed of at the site came from only one coal mine. The dumping facility is composed of two sections, namely the “old” one where the waste was disposed for several decades and the “new” one with the waste which came from the final stage of the mining operations at the coal mine (until 2012). Over the years, different technologies of coal enrichment were used in this coal mine, among others the flotation method. In the past, intense thermal phenomena were observed at this facility.

The third facility (III) was a functioning, partially reclaimed and partially burnt-out (exploited) extractive waste dump of an area equal to 56 ha. The estimated cubature of the site is about 25 million m^3^. Extractive waste has been deposited at the site since the first half of the twentieth century. The dump facility consists of the “old” section where burnt-out shale is extracted and the “new” section where extractive waste from the mine current production is disposed of. The expected height of the top surface of the facility is 350 m.a.s.l.

## Research methodology

The new, innovative sampling system applied in the study for the quantification of PAH compounds consisted of a sampling device with a solid polyurethane foam (PUF) sorbent, quartz fibre filter, an SKC Airchek2000 aspirator and a cooler cable. In the apparatus, the gases were collected at a flow rate of 3 L/min and 20 min sampling time. The adsorbed components were extracted by means of an Accelerated Solvent Extraction, and the DIONEX ASE 200 extractor with hexane. The obtained samples were analyzed using HPLC with a Fluorescence detector. In our study, a liquid chromatography method (using HPLC 1200 Series, Agilent Technologies) with a column ZORBAX Eclipse PAH (4.6 mm × 150 mm, 3.5 μm) was used. The mobile phase was a mixture of water/acetonitrile at 1.2 mL/min. The initial composition (50% acetonitrile) was held for 16 min and next increased to 100% over a period of 9 min, and then decreased to 50% over a period of 1 min. Validation Study Linearity was evaluated using standard solutions prepared by means of diluting the PAHs Mix standard solution in acetonitrile at six concentration levels (from 0.02 to 1 μg/L). Accuracy was evaluated in terms of recovery, which was determined for three replicates at two concentration levels (0.04 and 0.8 μg/L).

### Exploration method and experimental data modelling

The analysis of multidimensional experimental data was performed by means of a chemometrics method, in particular one of the most fundamental classic chemometrics methods which is the Hierarchical Clustering Analysis (HCA)^33–36^. The Hierarchical Clustering Analysis (HCA) allows analyzing the similarities/dissimilarities between objects in the parameters space and parameters in the object space, respectively. The data analyzed in the study was organized in matrix **X**(21 × 9) whose rows represent the studied objects, whereas the columns represent the measured averaged values of gaseous pollutants emitted to the atmosphere (see Table 1). In the case of the analyzed data, the samples representing averaged gaseous pollutants emitted to the atmosphere from the selected three dumps within the 21-month monitoring are the objects. The samples were averaged for particular quarters of the year (see Table 2).

**Table 1:** Parameters determined during the analysis of gaseous samples taken from the dumping facilities.

No.	Parameter	Unit
1	Naphthalene	µg/m^3^
2	Acenaphtene	µg/m^3^
3	Fluorene	µg/m^3^
4	Phenanthrene	µg/m^3^
5	Anthracene	µg/m^3^
6	Fluoranthene	µg/m^3^
7	Pyrene	µg/m^3^
8	B(a)anthracene	µg/m^3^
9	Chryzene	µg/m^3^

**Table 2:** The examined gaseous samples taken from the three dumping facilities (averaged for particular quarters of the year).

Sample no.	Dumping facility	Date of sampling
1	I	Quarter 3, 2017
2		Quarter 4, 2017
3		Quarter 1, 2018
4		Quarter 2, 2018
5		Quarter 3, 2018
6		Quarter 4, 2018
7		Quarter 1, 2019
8	II	Quarter 3, 2017
9		Quarter 4, 2017
10		Quarter 1, 2018
11		Quarter 2, 2018
12		Quarter 3, 2018
13		Quarter 4, 2018
14		Quarter 1, 2019
15	III	Quarter 3, 2017
16		Quarter 4, 2017
17		Quarter 1, 2018
18		Quarter 2, 2018
19		Quarter 3, 2018
20		Quarter 4, 2018
21		Quarter 1, 2019

The results of the Hierarchical Clustering Analysis (HCA) may differ depending on the applied measures of the similarities between the objects and the way in which similar objects are linked. In the case of continuous variables (such as those in this study), the most popular similarity measure—the Euclidean distance is used, whereas Ward’s algorithm is applied as the linkage method. Ward’s linkage method is defined as the sum of squared distances between each of the elements and the cluster. The results of the HCA are presented in the form of dendrograms; axis *x* describes the order in which the objects/parameters were linked, whereas axis *y* determines their similarity. The dendrograms allow analyzing the data structure, for example the data tendency to clustering. All chemometric analyses were carried out using Matlab software version 6.0 and author’s written routines^37^.

## Results and discussion

**Figure 1:**
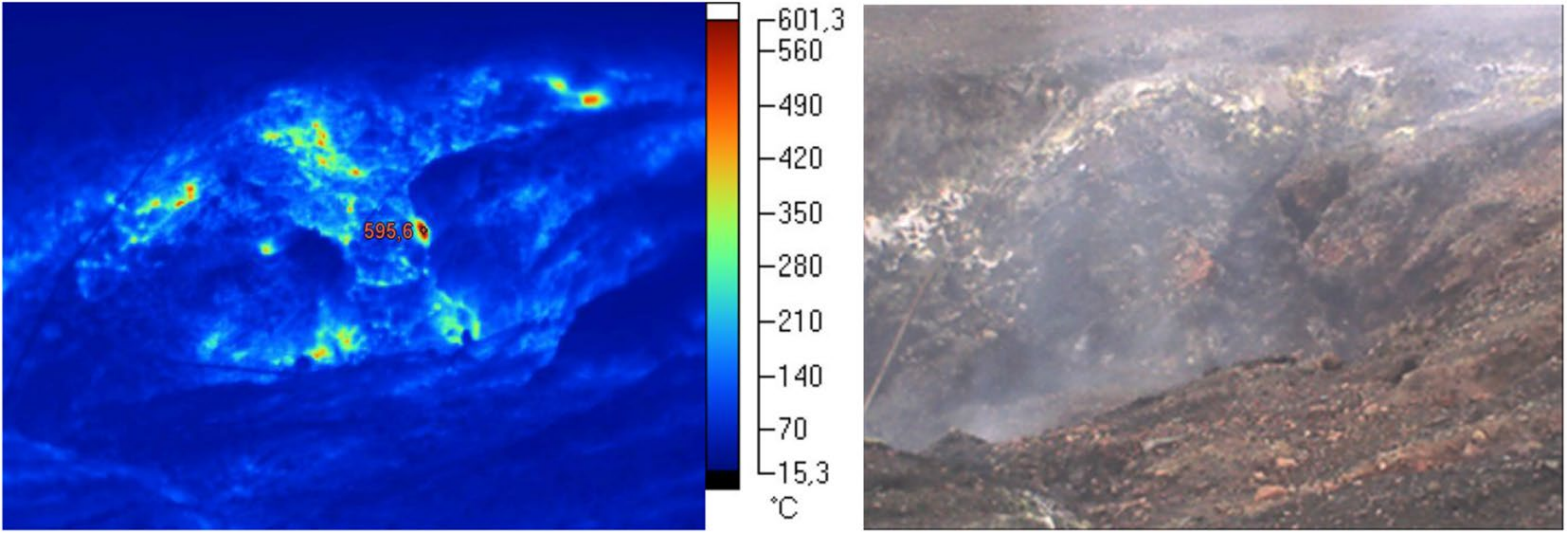
Thermo-visual examination of the extractive waste dump (Facility I); October, 2017.

**Figure 2:**
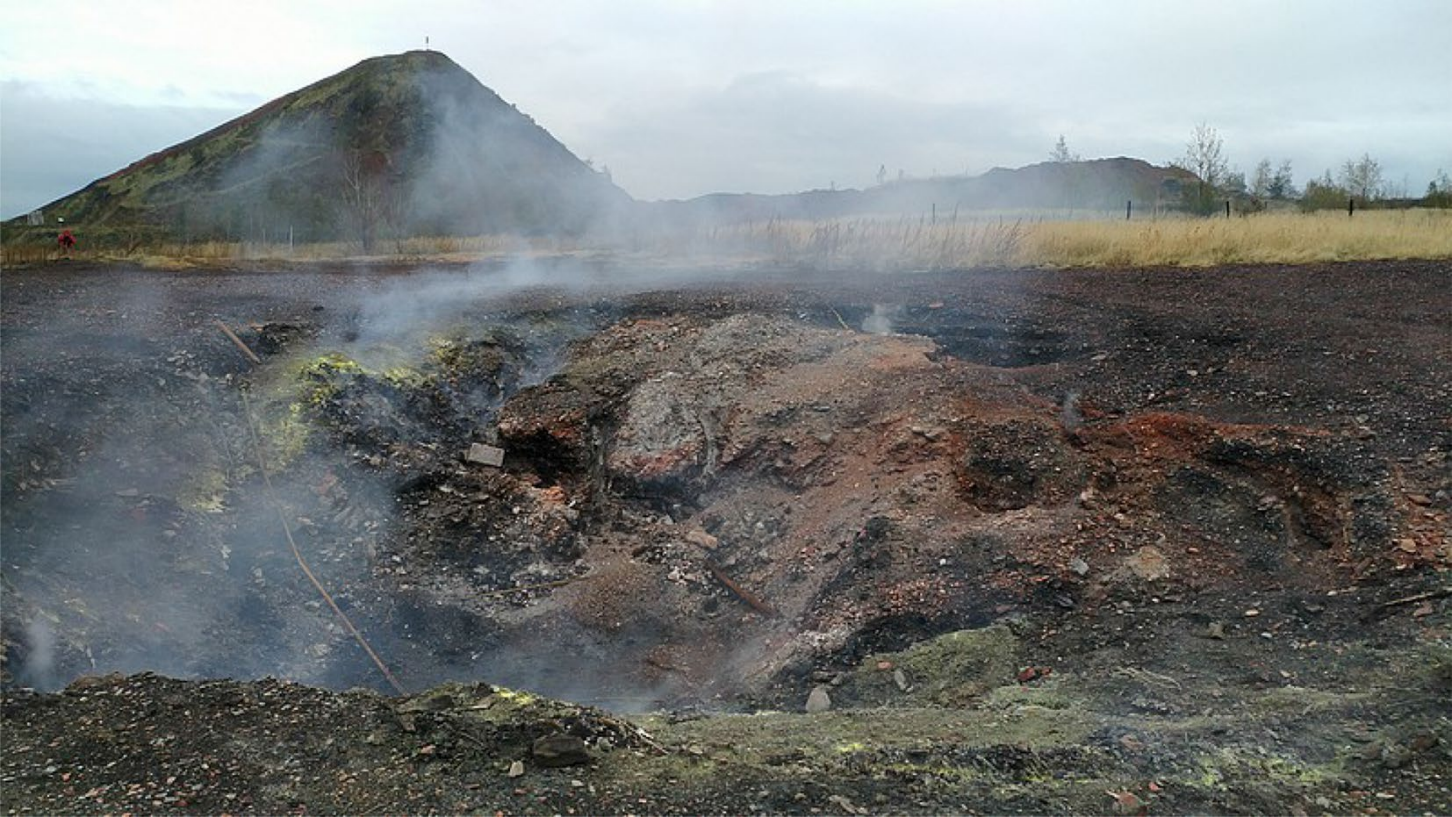
Subsidence (the so-called crater) at the top of Facility I; October, 2017.

In the case of the first studied facility—Facility (I), thermo-visual examinations did not demonstrate any signs of thermal activity in the majority of the area. Above all, the new layered sections of the facility are free from thermal phenomena. However, the central section of the dump is characterized by rather intense thermal phenomena. The cone is also thermally active, yet in this section the thermal activity is not very intensive (see Fig. 1). In the section with the strongest thermal activity, i.e. the southern part of the top, the recorded surface temperature exceeded 600 °C (see Fig. 2). Outside the thermally active zone, the surface temperature did not exceed 25–35 °C.

**Figure 3:**
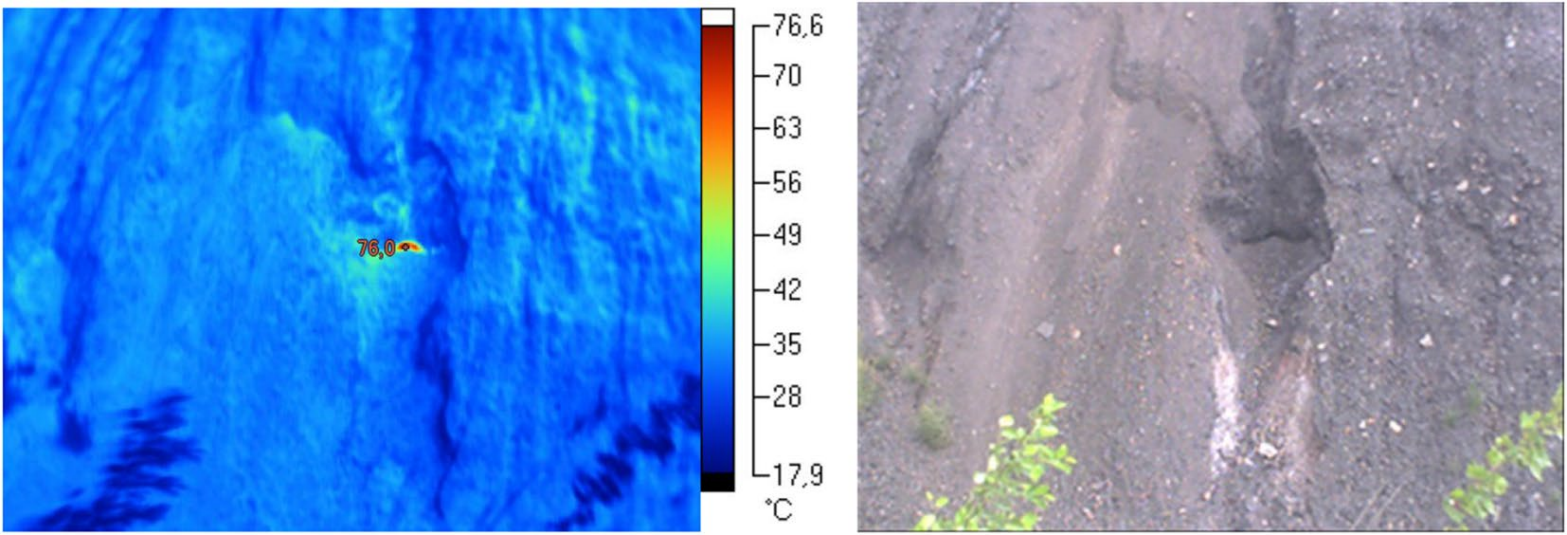
Thermo-visual examination of the extractive waste dump (Facility II); June, 2017.

The tests conducted on the premises of Facility II demonstrated the lack of thermal activity in the majority of the area. Only in the central section, on the top of the older part of the dump, the measurements showed the occurrence of thermal anomalies (see Fig. 3). These were minor areas located along the edge of the scarp on the northern and southern sides where the measured surface temperature was above 80 °C. It is worth mentioning that in 2010, the recorded temperatures for this section were at the level of several hundred degrees Celsius. Outside the sections, the temperature did not exceed 25–40 °C. The relatively high temperature in the area without thermal activity may be explained by the fact that in the morning hours it was exposed to the sun.

**Figure 4:**
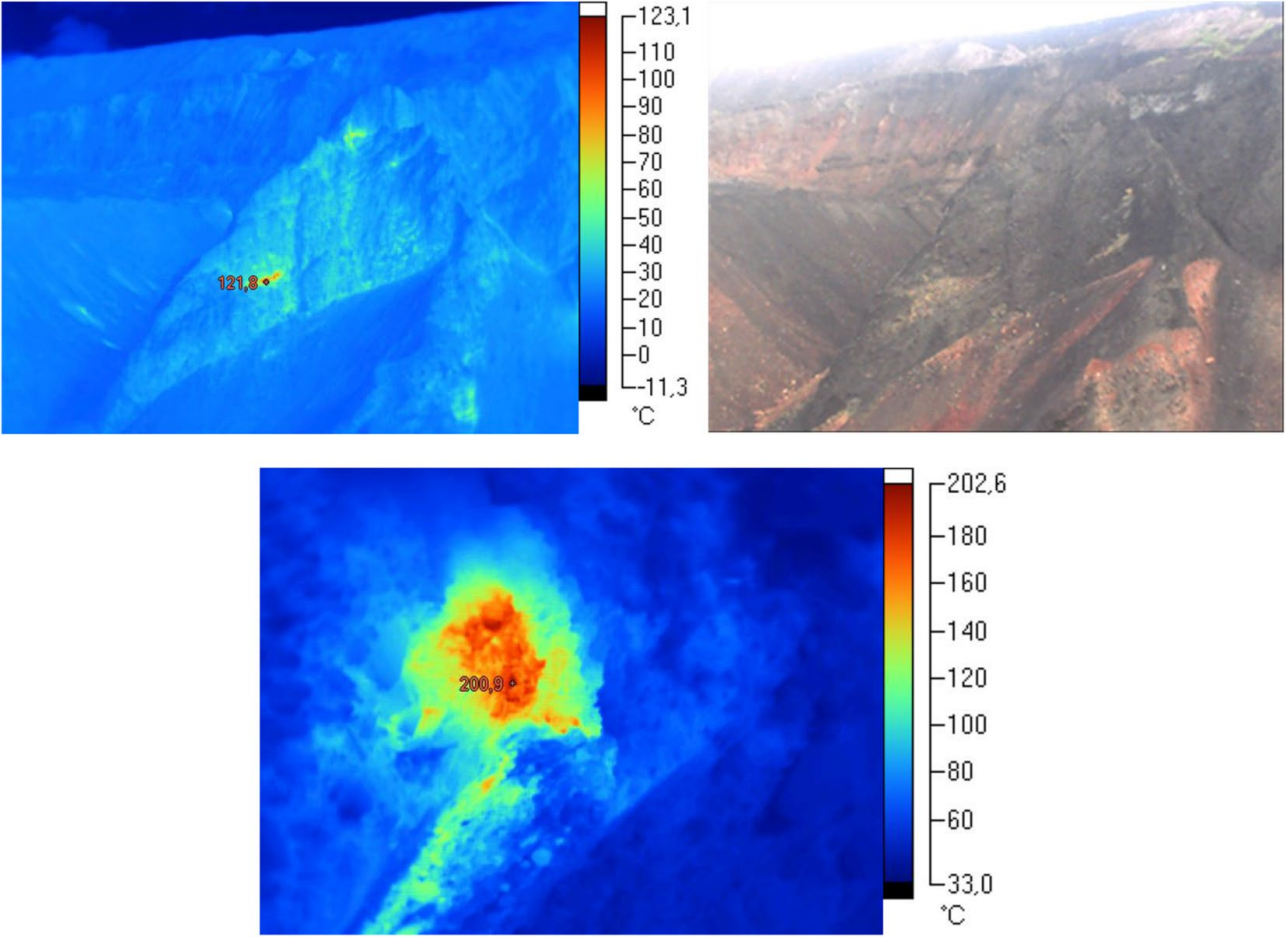
Thermo-visual examination of the extractive waste dump (Facility III); October, 2017.

During the course of the thermo-visual examination of the extractive waste dump in Facility III, no thermal activity was observed in the majority of the area. Only in the central section of the Facility, in the area which is still in operation, the measurements demonstrated the occurrence of thermal anomalies (see Fig. 4). These were minor areas where the local measured surface temperature was above 200 °C. Outside these areas, the temperature did not exceed 20–30 °C.

**Figure 5:**
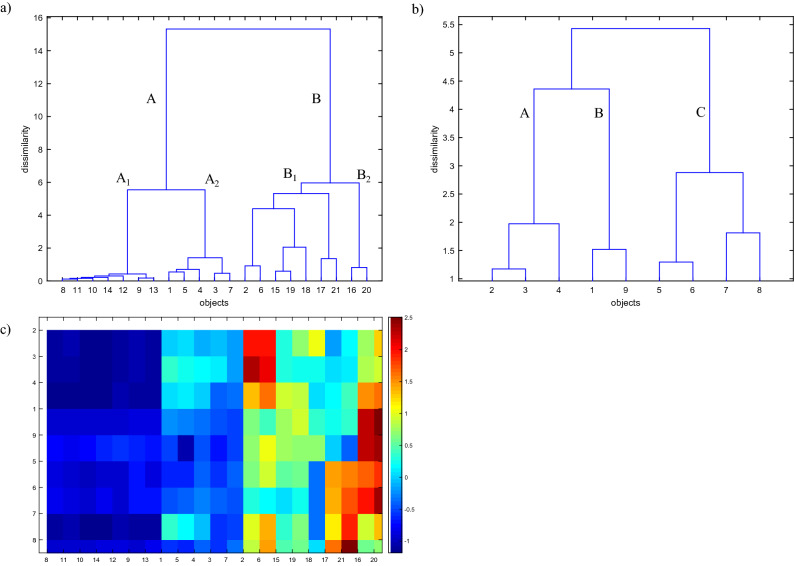
Dendrograms for (**a**) 21 objects representing the studied dumping facilities (see Table 1) in the space of 9 measured parameters; (**b**) parameters in the object space; (**c**) a color map presenting the values of measured parameters for particular dumping facilities.

The simplest method of analyzing the differences and similarities between the studied objects is their visualization in the space of measured parameters. When two or three parameters are measured, such visualization is not problematic. However, in our study there are nine measured parameters. The graphic presentation of a 9-dimensional space is not possible. This is the rationale of applying the HCA which enables to analyze the similarities between the studied objects (three different dumping facilities in different periods of time) as well as the similarities between the parameters in object space. Nevertheless, the HCA does not allow to simultaneously analyze the relationships between the objects and the measured parameters. This problem was solved by the use of a color map of the experimental data, which enabled an in-depth interpretation of the data structure. In addition, the application of the color map facilitates highlighting the differences and similarities among the clusters showed in the dendrograms, and, in consequence, it helps to distinguish the facilities which are characterized by the highest or the lowest values of the measured parameters. Figure 5 demonstrates the dendrogram for 21 objects representing the studied dumping facilities in different periods of time in the space of 9 measured parameters, the dendrogram for the measured parameters in the object space as well as a color map presenting the values of the measured parameters for particular objects.

Based on the dendrogram presented in Fig. 5a, a clear distinction of the examined objects representing the discussed dumping facilities into two clusters—A and B can be observed. Cluster A includes all samples representing Facility II in the whole period of the monitoring as well as samples representing Facility I taken in Quarter 3, 2017 and in Quarters 1–4, 2018 (objects nos. 1 and 3–6). All samples representing Facility III as well as two samples representing Facility I taken in Quarter 4, 2017 and in Quarter 4, 2018, respectively (objects nos. 2 and 6) are collected in Cluster B. In addition, within each of the clusters certain sub-clusters may be distinguished. Within Cluster A, the following sub-groups can be observed:

- sub-group A_1_ collecting all samples taken from Facility II during the whole period of the monitoring (objects nos. 8–14);
- sub-group A_2_ including samples taken from Facility I in Quarter III, 2017 and in Quarters 1–4, 2018 (objects nos. 1 and 3–6).
- In turn, Cluster B contains the following three sub-groups:
- sub-group B_1_ collecting two samples taken from Facility I in Quarter 4, 2017 and 2018, respectively (objects nos. 2 and 6) as well as samples representing Facility III in Quarter 3, 2017, Quarters 1–3, 2018 and Quarter 1, 2019 (objects nos. 15, 17–19 and 21);
- sub-group B_2_ encompassing the remaining two samples taken from Facility III in Quarter 4, 2017 and in Quarter 4, 2018 (objects nos. 16 and 20).
- The dendrogram obtained by means of Ward’s linkage method for the measured parameters in the space of 21 objects (see Fig. 5b) enables to distinguish the three principal clusters of parameters listed below:
- class A collecting parameters nos. 2, 3 and 4 (describing the concentrations of acenaphtene, fluorene and phenanthrene);
- class B containing parameters nos. 1 and 9 (describing the concentrations of naphthalene and chrysene);
- class C including the remaining parameters nos. 5, 6, 7 and 8 (describing the concentrations of anthracene, pyrene, fluoranthene and B(a)anthracene).

The PAHs emissions from a burning mine waste dump must be carefully monitored due to their potential toxicity and genotoxicity^38,39^. PAHs have two different roots; one is incomplete combustion of organic matter, whereas the other one is their production in the geological formation when organic sediments were chemically transformed into fossil fuels. In our study, the first path, namely spontaneous coal waste combustion is observed^40,41^. It is also worth mentioning that PAHs pose significant human health hazards. The exposure to PAHs may result in skin, lung or stomach cancers in the human organism^28^.

As mentioned before, a considerable drawback of the Hierarchical Clustering Analysis lies in that it does not allow for simultaneous interpretation of the dendrograms describing objects which represent particular samples taken from the extractive waste facility in different periods in the space of measured parameters and parameters in the object space. The lack of the possibility of interpreting the above relationships significantly limits the knowledge of the studied phenomena because the purpose of the analysis is to determine not only the differences among particular samples but also the underlying cause of such differences. Therefore, the dendrogram presenting the examined samples taken from the three selected facilities in different periods of time (see Fig. 5a) was juxtaposed with a color map of the experimental data (see Fig. 5c) demonstrating the values of the measured parameters arranged according to the order of the objects and parameters organization. The juxtaposition enables to determine the reason why the examined samples were distributed in such a way. In addition, the interpretation of the dendrogram for the objects in parameters space complemented with the color map of experimental data allows distinguishing samples which are characterized by the highest values of the measured parameters.

Analyzing the dendrogram presented in Fig. 5a together with the color map of the experimental data, it can be observed that all samples within Cluster A were characterized by relatively lower values of the measured parameters. Moreover, sub-group A_1_ identified within Cluster A was characterized by relatively lowest concentrations of acenaphtene, fluorene, phenanthrene and pyrene (parameters nos. 2, 3, 4, and 7) among all of the examined samples taken from the three selected facilities in the whole period of the monitoring. It confirms the observations which were made previously with the use of a thermo-visual camera which demonstrated that there were no thermal phenomena in the majority of the waste dump areas. The observed sparse emissions of gases result from certain thermal anomalies occurring in the central section of Facility II as well as some small areas located along the northern and southern side of the scarp; however, in any of the places the temperature did not exceed 80 °C (see Fig. 3). The thermo-visual examinations of the remaining sections of the facility showed that the surface temperature did not exceed the level of 25–40 °C and it is a result of sun exposure rather than the occurrence of any thermal phenomena.

Sub-group A_2_ which includes all samples taken from Facility I in Quarter 3, 2017 and Quarters 1–4, 2018 (objects nos. 1 and 3–6) differs from A_1_ objects in terms of relatively higher concentrations of acenaphtene, fluorene, phenanthrene and pyrene (parameters nos. 2, 3, 4, and 7). Additionally, within sub-group A_2_, the uniqueness of sample 1 taken in Quarter 3, 2018 (object no. 5) can be observed; it was characterized by the lowest concentration of chryzene (parameter no. 9) of all the examined samples.

Despite the fact that the results of the thermo-visual analysis of Facility III in general did not show any major signs of thermal activity, the Hierarchical Clustering Analysis by means of which the samples were examined in terms of the emission of the 9 parameters indicated that some thermal phenomena take place at this dump.

Furthermore, the analysis of the color map of the experimental data for the objects grouped in Cluster B makes it abundantly clear that thermal activity takes place in the majority of the monitored areas located at Facility III. What is more, for two samples taken from Facility I in Quarter 4, 2017 and Quarter 4, 2018 (objects nos. 2 and 6), thermal activity was observed. For the two objects, the concentrations of acenaphtene and fluorene are the highest among all of the examined samples (parameters nos. 2 and 3); similarly, the concentrations of phenanthrene, pyrene and B(a)anthracene (parameters nos. 4, 7, and 8) are also high. It confirms the observations made by means of a thermo-visual camera which demonstrated that there were no thermal phenomena if one considers the facility in its entirety. However, there are certain areas in the central section of the facility where thermal phenomena occur.

In the case of the samples taken from Facility III in Quarter 3, 2017 as well as in Quarter 2 and Quarter 3, 2018 (objects nos. 15, 18, and 19) within sub-group B_1_, the values of all measured parameters are only slightly increased, which can indicate the occurrence of thermal phenomena. Yet, it must be kept in mind that the concentrations are decidedly lower than for the remaining samples taken from Facility III. It may be also an indication that self-heating of the coal extractive waste takes place. The other two samples taken from Facility III in Quarter 1, 2018 and Quarter 1, 2019 (objects nos. 17 and 21) are characterized by relatively highest concentrations of pyrene and B(a)anthracene (parameters nos. 7 and 8) as well as high concentrations of anthracene and fluorene (parameters nos. 5 and 6), which can result from an intense thermal activity.

A similar observation can be made for the two samples taken from Facility III in Quarter 4, 2017 and Quarter 4, 2018 (objects nos. 16 and 20) which are characterized by the highest concentrations of naphthalene, anthracene, fluoranthene and chrysene of all the examined samples (parameters nos. 1, 5, 6 and 9) as well as high concentrations of phenanthrene (parameter no. 4). Although the results of the thermo-visual analysis of the whole area of Facility III did not demonstrate signs of thermal activity in most of its sections, there exist certain spots with intense thermal processes, which is confirmed by relatively high values of all the measured parameters.

## Conclusions

The new methodology of thermal activity monitoring at mine waste dumps was proposed. The correctness of the methodology was tested at three selected mine waste dumps. The research objective was to monitor the volumes of the emissions from the chosen extractive waste dumping facilities. The monitoring covered a period of 21 months. Based on the research findings, it was concluded that thermal phenomena occur at all of the three discussed facilities.

The crucial element of the developed methodology was the application of an advanced hierarchical clustering analysis to investigate the changes of atmospheric emissions of Polycyclic Aromatic Hydrocarbons from three extractive waste facilities located in the Upper Silesian Coal Basin, Poland.

The most intense thermal processes were observed on the premises of the non-reclaimed dumps and basically covered the majority of the studied area with surface temperatures reaching the level of 600 °C. In addition, the thermal phenomena were accompanied by noxious atmospheric emissions of Polycyclic Aromatic Hydrocarbons.

As concerns the partially reclaimed waste dump, some of its sections characterized of temperatures in the range of 20–30 °C, while the measurements confirmed atmospheric emissions of PAHs. It must be stated though that the phenomena were of lesser intensity than those occurring in the non-reclaimed facilities. In the case of the reclaimed dump, the highest measured temperature was 200 °C and high concentrations of the PAHs were observed only locally. In future research, the assessment of the volume of PAHs emissions may serve as a measure of the success rate concerning the reclamation process of extractive waste dumping facilities.
